# Sexual Assault: A Report on Human Immunodeficiency Virus Postexposure Prophylaxis

**DOI:** 10.1155/2010/196963

**Published:** 2010-07-29

**Authors:** William F. Griffith, Gary E. Ackerman, Cindy L. Zoellner, Jeanne S. Sheffield

**Affiliations:** ^1^Department of Obstetrics and Gynecology, University of Texas Southwestern Medical Center at Dallas, 5323 Harry Hines Boulevard, Dallas, TX 75390-9032, USA; ^2^Pharmacy Department, Parkland Health and Hospital System, Dallas, TX 75232, USA

## Abstract

The objective of this report is to describe an urban county hospital human immunodeficiency virus (HIV) infection prevention protocol offering prophylactic combination antiretroviral medications to female victims of sexual assault. A retrospective chart review was conducted from June, 2007 through June, 2008 of 151 women who were prescribed antiretroviral prophylaxis by protocol. All women receiving HIV prophylaxis initially screened HIV seronegative. Of the 58 women who reported taking any HIV prophylaxis, 36 (62%) were HIV screened at 12 and/or 24 weeks and none had HIV seroconverted. Although the initiation of an HIV post exposure prophylaxis protocol for sexual assault in a county hospital population is feasible, patient follow-up for counseling and HIV serostatus evaluation is an identified barrier

## 1. Introduction

The 2006 United States Census Bureau lists Dallas County, Texas, as a county of 2,345,815 individuals where approximately 1 of every 200 people is thought to be human immunodeficiency virus (HIV) seropositive [1, 2]. Human immunodeficiency virus prevalence among convicted sexual assault assailants may be twice the general male population which emphasizes the higher risk of HIV exposure following sexual assault [3]. 

Well-developed perinatal and postnatal HIV prophylaxis studies and the resultant protocols for reducing mother-to-child transmission include neonate prophylaxis which is believed to additionally protect the infant against infection from maternal HIV exposure during labor and delivery [4]. However, for ethical reasons and due to the inability to design a conclusive investigation, randomized and placebo controlled studies of sexual, nonoccupational postexposure prophylaxis (nPEP) will never be available. Data collected from an animal simian immunodeficiency virus transmission model in the macaque [5] and studies of health care workers receiving occupational postexposure prophylaxis [6] suggest that nPEP might reduce the risk for HIV infection after unanticipated sexual exposure. In an observational study of sexual assault survivors in Brazil, 180 women treated with combinations of zidovudine, lamivudine, and indinavir remained HIV seronegative while 4 (2.7%) of 145 untreated women seroconverted [7]. Although no study provides definitive evidence for the efficacy of sexual assault HIV nPEP, cumulative human, animal, and laboratory data demonstrate that timely antiretroviral therapy might reduce the risk for acquiring HIV infection. 

In 2005, the United States Department of Health and Human Services Centers for Disease Control (CDC) outlined recommendations for the provision of combination antiretroviral medications to prevent HIV infection after unanticipated sexual exposure [1]. Based on this recommendation, Parkland Health and Hospital System (PHHS) developed and implemented an HIV nPEP protocol to provide female sexual assault victims with standard health care guidelines for HIV-infection prevention. Our study presents a retrospective review of this protocol which provides high-risk sexual assault victims with antiretroviral prophylaxis medications.

## 2. Materials and Methods

In a collaborative PHHS effort that involved multiple disciplines including Obstetrics and Gynecology, Infectious Diseases, Patient Advocacy, and the Central Pharmacy, a Sexual Assault HIV Postexposure Prophylaxis Protocol (HIV nPEP) was designed. Using the CDC 2005 Guidelines for Antiretroviral Postexposure Prophylaxis after Non-occupational Exposure to HIV [1], the protocol was written to provide Noninfectious Disease specialists with clear assistance in victim counseling, in the prescribing of antiretroviral medications, and in arranging outpatient surveillance. The protocol also provided the framework from which cost underwriting for prophylaxis was linked to monetary assistance from the Texas State Crime Victims' Compensation Program (CVCP). 

Parkland Health and Hospital System is a large county hospital which provides emergency medical care to women and to Dallas County sexual assault victims through its Women's Intermediate Care Center (ICC). Upon arrival to PHHS, sexual assault victims aged 13 years and older are triaged for trauma which, if present, is immediately managed by the Trauma Emergency Department. Following trauma stabilization, the victim is then transferred to the ICC where specially trained rape counselors from the hospital's Victim Intervention Program/Rape Crisis (VIP) address emotional needs and assist with enrollment in the Texas CVCP. If the assault occurred within 72 hours and was either unprotected penis to vagina or rectum penetration, HIV nPEP labs are drawn. The University of Texas Southwestern Medical Center at Dallas (UTSW) Department of Obstetrics and Gynecology faculty provide all female forensic medical examinations and laboratory interpretations during which they screen the victim for high-risk HIV transmission elements. If the victim potentially qualifies for HIV nPEP, further counseling is provided by trained faculty outlining the risk of single-episode HIV transmission, the availability and known side effects of short-term antiretroviral prophylaxis, the necessary medication compliance required during the multidrug 28-day dosing regimen, and the need for further clinical evaluation and management. Once the victim understands counseling elements and agrees to receive HIV nPEP, prescriptions for combination antiretrovirals are provided which are initially filled without charge from the PHHS Central Pharmacy pending Texas CVCP funding. 

Because HIV nPEP after sexual assault remains an unproven clinical intervention, its provision should be offered on an individualized victim basis, but from standardized guidelines provided by a written protocol. The decision to proceed with HIV nPEP after a sexual encounter comes from the consideration of the five elements listed in Table 1 which trained faculty discuss in detail with the victim. The probability that the assailant is HIV infected is higher if the assailant uses illicit drugs, practices homosexual or bisexual activity, is a commercial sex worker, or if the assault occurs in a high-prevalence population. Antiretroviral prophylaxis is less likely to be effective if initiated later than 72 hours after HIV exposure. Medication compliance requires the patient to complete a 28-day course of multiple antiretroviral medications. A single episode risk of HIV transmission from a known infected source is dependent upon the exposure route (Table 2). Condom use during exposure reduces the exposure risk [8] whereas previous consensual, unprotected intercourse with the contact negates the benefit of prophylaxis.

Comprehensive patient counseling is integral to initiating any medical treatment that lacks a clearly proven benefit especially if the treatment has known side effects and toxicities. Antiretroviral medications selected for use in the PHHS HIV nPEP protocol include lopinavir/ritonavir (Kaletra) and emtricitabine/tenofovir (Truvada) or zidovudine/lamivudine (Combivir). Collectively, short-term side effects and toxicities for these medications may include gastrointestinal intolerance, asthenia, elevated transaminases, hyperglycemia, lipid abnormalities, bone marrow suppression, headache, insomnia, myopathy, hepatic steatosis, and renal impairment.

Medications selected for HIV prophylaxis depend upon the patient's age, pregnancy status, and laboratory test values. The HIV nPEP medication profile is detailed in Table 3. Assault victims qualified for HIV nPEP receive a baseline laboratory screen to ascertain the most appropriate HIV antiretroviral medication and dose to prescribe. Laboratory testing for the victim's initial HIV serostatus plus screening for the sexually transmitted diseases: Hepatitis B, Hepatitis C, syphilis, gonorrhea, and chlamydia are obtained. Any abnormal liver function and electrolyte test, creatinine clearance, or complete blood count prompts Infectious Disease or Pharmacy assistance in selecting the safest HIV medication and dose.

Continued care for sexual assault victims receiving HIV nPEP is provided by scheduled followup examinations at 2, 4, 12, and 24 weeks at a select PHHS Women's Clinic. During the victim's initial two-week followup, medication compliance and side effects are assessed and documented, ongoing ACA VIP counseling is scheduled as requested by the patient, and repeat labs, including liver function and electrolyte tests, creatinine clearance, and complete blood count, are evaluated. A UTSW Infectious Disease or HIV Pharmacist phone consult is obtained for abnormal lab results or for unmanageable medication side effects. Additional HIV serotesting is obtained at 4, 12, and 24 weeks postexposure. Seroconversion is reported following state and federal guidelines whereupon further patient care is arranged with an Infectious Disease specialist.

To evaluate the efficacy of the PHHS HIV nPEP protocol, medical charts of sexual assault victims receiving protocol driven antiretroviral prophylaxis were retrospectively reviewed with permission granted from the Institutional Review Board of the UTSW Medical Center at Dallas. Patients studied were female sexual assault victims from PHHS registered between June 18, 2007 and June 19, 2008.

Group comparisons were made using the chi-square test for categorical data and student's *t*-test for continuous measures. *P*-values less than 0.05 were considered statistically significant. All analyses were performed using SAS version 9.1 (SAS Institute, Cary, N.C.).

## 3. Results

The PHHS HIV nPEP protocol was initiated on June 18, 2007. In the first 12 months, we evaluated 660 sexual assault patients. One hundred and fifty-one received HIV nPEP medications. The demographic characteristics of these women are detailed in Table 4. The age of the women accepting HIV nPEP ranged from 13 to 61 years. Fourteen percent of the women resided outside Dallas County. The majority of women were brought to the hospital by emergency medical services or the Dallas County Police Department. Trauma services evaluated and stabilized 15 women (10%) for traumas external to the genital tract.

Baseline laboratory assessment was normal in 129 women (85%). Abnormal laboratory findings are listed in Table 5. No woman had a laboratory finding that affected the use of standard HIV nPEP medications. Baseline HIV and Hepatitis B serologic testing were negative in all women. Five women (3%) were Hepatitis C positive but all had normal liver function testing. One woman was pregnant and was started on lamivudine/zidovudine and lopinavir/ritonavir per protocol. Two (1%) women were diagnosed with syphilis, 17 (11%) women with Chlamydia, and 7 (5%) with gonococcal cervicitis at the time of initial evaluation which reflects infection prevalence in the ICC emergency care population.

There are multiple known drug interactions associated with antiretroviral medications. Thirty-four women (23%) were taking at least one medication known to have potential drug interactions with protocol antiretrovirals, mainly psychiatric and oral contraceptive medications. These women were counseled regarding the potential drug interactions and possible measures to prevent complications, for example, condom use to prevent pregnancy during the HIV nPEP time period.

Sixty-two women (41%) presented to the hospital system for followup during the study period, where 58 (94%) of these women self-reported taking any HIV nPEP medication. Thirty-seven women (60%) reported taking a 21-day HIV nPEP or completing the prescribed course of therapy. Women presenting for the first surveillance appointment at 2 weeks were significantly more likely to complete subsequent followup visits (*P* < .02). We did note an increase in followup rates from the first 3 months of the protocol compared to the last 3 months (38% and 46%, resp.), probably reflecting increased provider familiarity with the protocol and an associated improvement in patient counseling and education. Nineteen women had one followup visit, 12 had 2 visits and 31 women completed 3 or more of the scheduled visits. Women presenting for any followup were significantly younger where 60% of women <18 years of age had at least one followup visit (Table 6). There were no significant differences in race or county of residence with regards to followup.

No woman seroconverted for Hepatitis B or C nor were there new cases of syphilis identified. Although there were no HIV seroconversions during our study, only 15 women received an HIV test ≥6 months after receiving HIV nPEP. 

Five women (8%) who presented for followup had evidence of laboratory toxicity from the HIV nPEP medications. One woman receiving zidovudine developed neutropenia with an absolute neutrophil count decreasing from 3260 to 1200 cells/mm^3^. One woman had an increase in serum creatinine from 0.87 mg/mL baseline to 1.22 mg/mL at 2 weeks, and 3 women had evidence of worsening liver function though only one required the HIV nPEP medications to be stopped. Side effects were common; 67% of women reported at least one side effect with gastrointestinal complaints being the majority. One woman had an allergic reaction though we were unable to determine which of her medications was the source (pain, antibiotic, or HIV nPEP medications). Nine women reported using prescription or over-the-counter medication to treat side effects, but only four stopped HIV nPEP secondary to symptoms. The majority of women who received followup tolerated the HIV nPEP medications and were able to complete the 28-day course.

## 4. Discussion

Definitive published data on the efficacy of HIV nPEP among those sexually assaulted is lacking. Thus, the decision to offer combination antiretroviral medication is based on the rate of infection incurred through different types of sexual intercourse plus the suspected prevalence of HIV infection among assailants. Undeniably as important as infection rate statistics, sexual assault victims often express significant anxiety from the fear of contracting an HIV infection even though the chances of HIV transmission following a single sexual contact are very low. Data shows immediately after HIV infection but before a host develops an immune response, HIV particles replicate and create more than 10 billion new virions per day [9]. With timely combination antiretroviral therapy, a 50% reduction in viral load can be seen by day 3 and, thereafter, a further reduction by more than 99% [10]. Therefore, the possibility of preventing HIV transmission by prophylaxis should be considered according to current CDC guidelines [1].

Determining the individual victims risk for infection is initially based upon five key elements (Table 1). However, many of our sexual assault victims are self-identified sex workers, current substance abusers, or claimers of previous consensual sex with the assailant, all of which amplify the overall life-time risk of HIV infection. Many victims are unwilling to assure medication compliance or decline the offer of antiretroviral prophylaxis after counseling, accepting the low-risk of HIV transmission instead. These various victims' reasons as data points for not being offered or for refusing HIV nPEP were not collected during this retrospective review but will be explored in a future investigation.

The Texas Association Against Sexual Assault in a 2003 survey of emergency rooms providing care for sexual assault victims found that 70% of respondents offer and discuss treatment and testing options for HIV [11]. However, many health care facilities lack a well-defined protocol for the treatment and surveillance of these patients. The implementation of such a protocol in the general population can be difficult and costly, and data are sparse regarding issues related to such implementation. Even though this paper illustrates the feasibility of such a protocol, medication compliance and postassault surveillance remain unclear but complicated barriers to successful HIV nPEP. With patient consent, possible solutions may involve a more supportive case management approach through frequent telephone or online communications.

The main strength of the PHHS HIV nPEP protocol comes from the inclusive management of sexual assault victims within one coordinated hospital setting. From forensics evidence collection and VIP rape victim advocacy counseling to the actual provision of prescription medications with financial assistance, our protocol guideline supports continuity of care. Patient followup is provided at a single, easily accessible PHHS Women's Clinic by providers trained to manage sexual assault patients. Ongoing identification of health care inconsistencies that require improvement is more efficient within a single health care delivery system. 

One of the greatest hurdles in implementing this type of protocol is cost. The average cost of combination antiretroviral medication for 28 days of prophylaxis is approximately $1,000 resulting from public health system outpatient pricing at PHHS. Therefore, simply offering costly prophylactic medication is insufficient unless funding arrangements allow immediate drug access. Through a collaborative effort involving PHHS's Central Pharmacy, Departments of Obstetrics and Gynecology and Internal Medicine/Infectious Diseases Division, and VIP counseling, high-risk assault victims can be given HIV nPEP medications underwritten by the hospital but linked to payment by the Texas CVCP [12]. To accomplish this, the PHHS VIP provides comprehensive counseling for sexual assault victims that includes assistance with the CVCP application. Completion of the CVCP application prompts the Central Pharmacy to dispense HIV nPEP medications to the patient at no initial charge. The total cost of prescribing and underwriting this medication for our 151 high-risk sexual assault patients was approximately $160,000, a figure substantially less than the $385,000 monetary lifetime cost of care for one HIV seroconversion [13]. Crime Victims' Compensation Program funding opportunities, such as that in Texas, are also available in most states and should be made readily accessible to sexual assault victims.

The main weakness of this retrospective review was our inability to track patient HIV seroconversion beyond our surveillance schedule due to the lack of patient consent. Furthermore, determining if an HIV seroconversion at a later date was due to an infection received during a sexual assault and failure of HIV nPEP or from resumed consensual sexual behavior would be difficult. Although there were no HIV seroconversions in our study group, only 15 women received an HIV test at a 6-month or later screen. Prophylactic medication full compliance and patient followup counseling remain ongoing protocol issues most likely related to the challenges providers have in delivering detailed HIV nPEP information to the emotionally traumatized sexual assault patient. Patient followup in general improved during the last 3 months of the study period probably due to increased provider familiarity with the protocol resulting in enhanced patient education. Overall, followup in our system was 41%, a figure higher than that previously reported in [14], and self-reported medication compliance was good with 60% of women stating that they took 21 days or completed their HIV PEP regimen at their four-week followup appointment. Gastrointestinal side effects were frequent but rarely prevented completion of HIV PEP therapy. There were no unanticipated adverse events.

Human immunodeficiency virus nPEP counseling that promotes informed patients decision making should be offered to all eligible high-risk sexual assault patients. Immediate medication accessibility and funding are equally important if the timely provision of prophylaxis is to be achieved. A well-defined protocol with continuity of care provided by a single health care system encourages standard, high-quality patient management. Individual hospital systems can successfully adapt the 2005 CDC recommendations for antiretroviral prophylaxis after nonconsensual sexual exposure by giving specific attention to medication funding and patient surveillance practices.

## Figures and Tables

**Table 1:** Key elements to consider prior to HIV postexposure prophylaxis.

(i) Probability that the assailant(s) is HIV infected
(ii) Treatment initiated within 24 not to exceed 72 hours after exposure
(iii) Victim ability to comply with drugs as prescribed
(iv) Risk of HIV transmission by a particular exposure
(v) Condom use during exposure reduces exposure risk

Adapted from the CDC MMWR 2005 [1].

**Table 2:** HIV transmission risk from known HIV seropositive exposure.

(i) Blood transfusion: 9,000 per 10,000 exposures
(ii) Needles sharing injection drug use: 67 per 10,000 exposures
(iii) Receptive anal intercourse: 50 per 10,000 exposures
(iv) Percutaneous needle stick: 30 per 10,000 exposures
(v) Receptive penile-vaginal intercourse: 10 per 10,000 exposures
(vi) Receptive oral intercourse: 1 per 10,000 exposures

Adapted from CDC MMWR 2005 [1].

**Table 3:** HIV nPEP antiviral medications doses.

(1) If the patient is ≥18 years of age with a negative urine pregnancy test, normal liver function and complete blood count tests, and a creatinine clearance of ≥50 mL/minute:	
Emtricitabine/tenofovir: one tablet by mouth every day for twenty-eight days	
PLUS	
Lopinavir/ritonivir: two tablets by mouth twice daily for twenty-eight days	

(2) If the patient is ≥12 and <18 years of age or is pregnant at any age with normal liver function and complete blood count tests and a creatinine clearance of ≥50 mL/minute:	
Lamivudine/zidovudine: one tablet by mouth twice daily for twenty-eight days	
PLUS	
Lopinavir/ritonivir: two tablets by mouth twice daily for twenty-eight days	

(3) Patients with Hepatitis B or C may be given emtricitabine/tenofovir and lopinavir/ritonivir as long as their baseline liver function tests are normal.	

(4) Patients with baseline abnormal renal function should not receive emtricitabine/tenofovir. Instead, they should be started on lopinavir/ritonavir (as above) plus lamivudine/zidovudine with dosing adjustments listed below.	

Dosage adjustments for patients ≥12 years of age with CrCL <50 mL/minute	
CrCL	Renal dosage

30–49 mL/minute	Lamivudine 150 mg tablet QDay zidovudine 300 mg tablet BID
15–29 mL/minute	Lamivudine oral solution 100 mg/10 mL QDay zidovudine 300 mg tablet BID
5–14 mL/minute	Lamivudine oral solution 50 mg/ 5mL QDay zidovudine 300 mg tablet BID
<5 mL/minute	Lamivudine oral solution 25 mg/2.5 mL QDayzidovudine 300 mg tablet QDay

Adapted from CDC MMWR 2005 [1].

**Table 4:** Characteristics of the women receiving HIV nPEP medications.

	Cohort *N* = 151
Age (mean years ± SD)	30 ± 11
<18 years of age	43 (28)
Range (years)	13–61
Race	
African-American	57 (38)
Caucasian	57 (38)
Hispanic	37 (24)
County of residence	
Dallas	130 (86)
Out of county (TX)	18 (12)
Out of state	3 (2)
Mode of transport to the hospital	
Emergency medical services	16 (11)
Police	69 (46)
Self	64 (42)
Unknown	2 (1)
Trauma service evaluation	15 (10)

Data presented as *n* (%) unless otherwise noted.

**Table 5:** Baseline laboratory evaluation prior to receiving HIV nPEP medications.

	Cohort *N* = 151
Liver transaminases ≥2 SD	0 (0)
Hematocrit <35%	17 (11)
Hematocrit <30%	3 (2)
Thrombocytopenia (150,000 platelets)	0 (0)
Glucose >200 mg%	2 (1)
Hypophosphatemia	1 (1)
Creatinine >1.2 mg/mL	0 (0)
Hepatitis C seropositive	5 (3)
HIV seropositive	0 (0)
Hepatitis B surface antigen	0 (0)

**Table 6:** Comparison of women who presented for followup to those with no followup.

	Any Followup *N* = 62	No Followup *N* = 89	*P*-value
Mean age	22.7 ± 9.9	26.6 ± 11.5	.03
<18 years of age	26 (42)	17 (19)	<.002

Race			
African American	19 (31)	38 (43)	NS
Caucasian	23 (37)	34 (38)	
Hispanic	20 (32)	17 (19)	

County of residence			
Dallas	54 (87)	76 (85)	NS
Out of county	8 (13)	10 (11)	
Out of state	0	3 (3)	
